# Computer-Aided Screening of Autism Spectrum Disorder: Eye-Tracking Study Using Data Visualization and Deep Learning

**DOI:** 10.2196/27706

**Published:** 2021-10-25

**Authors:** Federica Cilia, Romuald Carette, Mahmoud Elbattah, Gilles Dequen, Jean-Luc Guérin, Jérôme Bosche, Luc Vandromme, Barbara Le Driant

**Affiliations:** 1 UR-UPJV 7273 Centre de Recherche en Psychologie - Cognition, Psychisme, Organisations Université de Picardie Jules Verne Amiens France; 2 UR-UPJV 4290 Modélisation, Information & Systèmes Université de Picardie Jules Verne Amiens France; 3 UR-UPJV 7516 Chirurgie et Extrémité Céphalique Caractérisation Morphologique et Fonctionnelle Université de Picardie Jules Verne Amiens France

**Keywords:** autism spectrum disorder, screening, eye tracking, data visualization, machine learning, deep learning, AI, ASS, artificial intelligence, ML, screening, adolescent, diagnosis

## Abstract

**Background:**

The early diagnosis of autism spectrum disorder (ASD) is highly desirable but remains a challenging task, which requires a set of cognitive tests and hours of clinical examinations. In addition, variations of such symptoms exist, which can make the identification of ASD even more difficult. Although diagnosis tests are largely developed by experts, they are still subject to human bias. In this respect, computer-assisted technologies can play a key role in supporting the screening process.

**Objective:**

This paper follows on the path of using eye tracking as an integrated part of screening assessment in ASD based on the characteristic elements of the eye gaze. This study adds to the mounting efforts in using eye tracking technology to support the process of ASD screening

**Methods:**

The proposed approach basically aims to integrate eye tracking with visualization and machine learning. A group of 59 school-aged participants took part in the study. The participants were invited to watch a set of age-appropriate photographs and videos related to social cognition. Initially, eye-tracking scanpaths were transformed into a visual representation as a set of images. Subsequently, a convolutional neural network was trained to perform the image classification task.

**Results:**

The experimental results demonstrated that the visual representation could simplify the diagnostic task and also attained high accuracy. Specifically, the convolutional neural network model could achieve a promising classification accuracy. This largely suggests that visualizations could successfully encode the information of gaze motion and its underlying dynamics. Further, we explored possible correlations between the autism severity and the dynamics of eye movement based on the maximal information coefficient. The findings primarily show that the combination of eye tracking, visualization, and machine learning have strong potential in developing an objective tool to assist in the screening of ASD.

**Conclusions:**

Broadly speaking, the approach we propose could be transferable to screening for other disorders, particularly neurodevelopmental disorders.

## Introduction

### ASD Characteristics

Autism spectrum disorder (ASD) has been described as a pervasive developmental disorder characterized by a set of impairments including social communication problems, difficulties with reciprocal social interactions, and unusual patterns of repetitive behaviors or interests [1]. During naturalistic interaction, making and maintaining eye contact is not always easy or spontaneous for ASD-diagnosed individuals. Such troubling deficits can unfortunately place a considerable strain on their lives and their families. Nevertheless, these disturbances are not better explained by intellectual disability or global developmental delay [1].

Early diagnosis may lead to early intervention, which generally proves beneficial for both the child and the family. The diagnosis process usually involves a set of tests that can require hours of clinical examinations or is based on an interview with the parents. Furthermore, the variation of symptoms with regard to deficits in social communication and social interaction as well as the social communication impairments and restricted, repetitive patterns of behavior make the identification of ASD more complicated to decide. In this respect, computer-aided technologies have been embraced to provide helpful guidance through the course of examination and assessment. Examples include magnetic resonance imaging, electroencephalography [2], and eye tracking, which will be considered in this study. Eye-tracking technology has received particular attention in the ASD context since abnormalities of eye gaze have been consistently recognized as the hallmark of autism in general [3,4]. A considerable number of other psychology studies in eye tracking have been based on the particularities of eye movements in response to verbal or visual cues as signs of ASD [5-7]. In particular, these studies have highlighted social-related difficulties in children with ASD, especially when face stimuli are used (eg, in a face-to-butterfly categorical visual search task [8] and unsuitable extraction of visual information via eye fixations for emotion recognition [9]).

This study provides a meeting point for eye tracking and machine learning (ML) for supporting the diagnosis of ASD. It is part of an interdisciplinary collaboration between research units of psychology and artificial intelligence at the University of Picardy Jules Verne in France. Our approach is distinctively based on the premise that visual representations of eye-tracking recordings can effectively serve as features for discriminating individuals diagnosed with ASD. At its core, the key idea is to compactly render eye movements into an image-based format while maintaining the dynamic characteristics of eye motion (eg, velocity) using color gradients. In this manner, diagnostic-related tasks can be approached as a problem of image classification or analysis. The applicability of the proposed approach will be demonstrated based on the classification accuracy. Further, we will support our results with a statistical analysis that will explore possible correlations between the Childhood Autism Rating Scale (CARS) [10] and the dynamics of eye movement among participants diagnosed with ASD.

### Eye Tracking for ASD Screening

Eye tracking has been used in numerous research studies. It can be described as the process of capturing, tracking, and measuring eye movements or the absolute point of gaze (POG), which refers to the point where the eye gaze is focused in the visual scene [11]. The significance of such technology is that it allows for an objective and quantitative method of recording the viewer's POG. The interpretation of eye movement can be effectively used in interactive applications or for diagnostic purposes.

Eye trackers aim to capture 3 basic categories of eye movements: (1) fixation, (2) saccade, and (3) blink. A fixation is the brief moment that occurs while pausing the gaze on an object so that the brain can perform the perception process. The average duration of fixation typically ranges from 150 ms to 300 ms [12]. However, the fixation duration is dependent on the context. The duration of our fixations differs when we are reading on paper (230 ms) or on a screen (553 ms) [13], or when we are watching a naturalistic scene on a computer (330 ms) [14]. Further, accurate perception requires constant scanning of the object with rapid eye movements, which are called saccades. Saccades include quick, ballistic jumps that take about 30-120 ms each [15]. On the other hand, a blink is often a sign that the system has lost track of the eye gaze. Eye-tracking scanpaths have been commonly used as a practical means for depicting gaze behavior in a visual manner. A scanpath represents a sequence of consecutive fixations and saccades as a trace through time and space and may overlap with itself [16].

Abundant studies have sought to take advantage of eye-tracking applications for studying and analyzing eyes movements. For instance, a team of psychologists and neuroscientists recently showed that children with ASD have faster eye movements than do children with typical development, but these results depend on the visual task the children are asked to perform. If they are faster while remaining precise in prosaccade tasks with a gap paradigm, the same children are less accurate but faster than are children with typical development in another gap paradigm during short visual search. This means that children with ASD favor speed over accuracy and that they have shorter saccadic latencies [8]. Moreover, Vabalas and Freeth [17] demonstrated that in face-to-face interactions, eye movements were different among individuals depending on where they fell on the autism spectrum. Specifically, persons with high autistic traits were observed to experience shorter and less frequent saccades. Conversely, Liberati et al [18] showed greater saccade amplitude and higher frequency in children with ASD than did control children. However, in Liberati et al’s study, the Tobii Eye Tracker used had a sampling rate of 60 Hz, and they extracted the raw data to create clusters using k-means clustering, whereas Vabalas and Freeth used SMI eye-tracking glasses with a rate of 24 Hz. Thus, the question does not appear to be settled and seems to depend largely on the equipment and data used (eg, autistic traits according to a questionnaire vs autistic persons). In another study, eye-tracking was used to identify children diagnosed with ASD based on the duration of fixations and the number of saccades [19]. The results showed that participants with ASD spent significantly more time fixating on dynamic geometric images compared to other diagnostic groups. Likewise, a longitudinal study examined the fixation patterns of infants from 2 to 6 months of age [20]. It was found that infants diagnosed with ASD exhibited a mean decline in fixations, which was not observed for those who did not develop ASD afterwards. Moreover, another cohort study suggested the strong potential of eye tracking as an objective tool for quantifying the risk of autism and estimating the severity of its symptoms [21]. A high diagnostic accuracy was demonstrated in this regard as well.

### ML for ASD Screening

ML is subfield of computer science involved in providing computers the ability to learn without being explicitly programmed [22]. In contrast to traditional programming, ML attempts to extrapolate algorithms from data exclusively. Thus, the power of ML is that it allows for extracting insights, making predictions, or taking actions with minimal human intervention (if any). The development of ML can be broadly organized into supervised or unsupervised models. On the one hand, supervised ML deals with labeled examples, where the desired output is known precisely. The learning algorithm receives a set of inputs along with corresponding labels, and the algorithm can learn by comparing predicted labels to the actual ones. The model can be iteratively optimized to minimize error. On the other hand, unsupervised ML uses training data that do not include any output information (ie, labels). Unsupervised models (eg, clustering and association rules) can provide descriptive knowledge to help understand the inherent structure or properties of the data.

The coupling of eye tracking with ML is currently leveraging further capabilities for advancing ASD diagnosis and its applications. The literature includes several contributions in this context. For instance, Pusiol et al [23] worked on the analysis of the eye focus on the face during conversations. Their analysis was specifically applied to children with developmental disorders or those with fragile X syndrome. They tested a set of classification models, including recurrent neural networks, support vector machine, Naive Bayes, and the hidden Markov model. With recurrent neural networks, they were able to reach a high prediction accuracy of 86% and 91% for the classification of female and male fragile X syndrome, respectively. Another recent study applied ML on eye-tracking output to predict ASD [24]. The ML model included features related to the saccade eye movement (eg, amplitude, duration, and acceleration). The experiments were aimed at detecting ASD among a set of 17 children aged 8 to 10 years. Despite the use of a limited data set and a relatively simple model, the findings demonstrated the promising potential of ML for this application.

Other recent studies have focused on predicting the visual attention of children with ASD. For instance, Wei et al [25] proposed a saliency prediction model based on a convolutional neural network (CNN), but they concluded that it is necessary to first train the model on an eye-tracking data set of typical development to enable more effective saliency prediction. Jiang et al [26] proposed a method with 86% accuracy that classifies eye fixations based on a comprehensive set of features and that integrates task performance, gaze information, and facial features extracted using a deep neural network. Their work focused on a population of children with ASD between the ages of 8 and 17 years whose intellectual level was highly disparate (IQ score range 58-137).

Compared to the literature, the main distinction of this paper is that it is purely reliant on the visual representation of eye-tracking scanpaths. The study aims to produce scanpath visualizations that can represent the spatial patterns of gaze behavior and its dynamics. In this way, the vision-based approach allows for approaching the diagnosis problem as a typical task of image classification and is a continuation of our earlier work [24,27]. Our initial work applied a different set of features based on the events of fixations and saccades. We have transformed the eye-tracking data into a visual representation [27,28]. This study builds on our earlier efforts in an attempt to develop more sophisticated ML models using deep learning.

## Methods

### Recruitment

A group of 59 children took part in this study. It was highly desirable to have participants at an early stage of development, as the principal goal was supporting the early detection and diagnosis of ASD. Specifically, all participants were school-aged children of a mean age of about 8 years. This somewhat advanced age was indispensable here because in our region there were not enough diagnosed children younger than 6 years, and the time it takes to consult a doctor to make a diagnosis can be as long as 2 years. For the group of typically developing children (non-ASD), parental reports of any possible concerns were carefully considered.

The ASD diagnosis was confirmed by health professionals using standardized tools (Autism Diagnostic Interview-Revised [ADI-R], and Autism Diagnostic Observation Schedule–Generic [ADOS-G]). However, we did not get permission to read the children’s medical files. ADI-R and ADOS-G scores were not analyzed in this study. The participants were broadly organized into 2 groups: (1) diagnosed with ASD or (2) non-ASD. Children diagnosed with ASD were examined in multidisciplinary ASD specialty clinics. The intensity of autism was estimated by psychologists using the French version of the CARS [29], while communication level was assessed with the French version of the Early Social Communication Scale (Echelle d’évaluation de la Communication Sociale Précoce [ECSP]) [30]. Table 1 summarizes the statistics of the participants.

All the children’s parents or legal guardians were informed of the objectives of the study, the nature of the tasks that would be administered, and the fact that they could withdraw their agreement at any time. Their informed consent was received in writing in accordance with the Declaration of Helsinki of June 1964 (amended at the 64th General Assembly of the World Health Organization in October 2013). Moreover, all children gave their agreement to participate, and if they wished, parents could be present with their children in the experimental room. This study did not require authorization from an ethics committee based on the recommendations for psychological research in France and in agreement with the national and institutional guidelines.

**Table 1:** Summary of participant statistics.

Child group	ASD^a^ (n=29)	Non-ASD (n=30)
Males, n	19	19
Chronological age (years, months), mean (SD)	7, 7 (2, 6)	8 (2, 8)
Developmental age on the ESCS^b^ (months, days), mean (SD)	24, 10 (6, 8)	23, 15 (6, 7)
Total ECSP score, mean (SD)	141, 1 (50, 3)	139, 18 (49, 5)
CARS^c^ score (minimum score=15; autism cutoff > 30), mean (SD)	32, 9 (6, 4)	15 (0)

^a^ASD: autism spectrum disorder.

^b^ECSP: Echelle d’évaluation de la Communication Sociale Précoce.

^c^CARS: Childhood Autism Rating Scale.

### Apparatus and Stimuli

The SMI RED250 remote eye tracker (250 Hz, SensoMotoric Instruments) was the main instrument used to perform the eye-tracking function. The device belongs to the category of screen-based eye trackers. It can be conveniently placed at the bottom of the screen of a desktop PC or laptop. In our case, a 17-inch monitor with a 1280 x 1024 resolution was used.

Further stimuli were presented from the SMI Experiment Center software. Stimuli represented multiple distinct types used in the eye gaze literature. Examples included static and dynamic naturalistic scenes with and without receptive language, joint attention stimuli, static face or objects, and cartoon stimuli. The average duration of eye-tracking experiments was about 10 minutes. Participants were mainly examined for the quality of eye contact with the presenter and the level of focus on other elements. A 5-point calibration scheme was used. The calibration routine was followed by a set of verification procedures.

### Procedure

The participants were invited to watch a set of photographs and videos, which included scenarios tailored specifically to stimulate the eye movement across the screen area. Participants could be seated on their own or on their parents’ lap at an approximately 60-cm distance from the display screen. The experiments were conducted in a quiet room at the university premises. Physical white barriers were also used to reduce visual distraction.

The scenarios varied in content and length in order to allow for analysis of the ocular activity of participants from different perspectives. In general, videos were designed to include visual elements that are especially attractive to children (eg, colorful balloons and cartoons). Specifically, the stimuli presented are part of various psychological studies. One of these studies involves the presentation of 3 videos including a situation of joint attention initiation (duration of 58 seconds per video) and 18 photos from the same situation (5 seconds per photo). The scene presented in the video and corresponding photos started with an attention grabber (ie, a hand-waving cartoon). The woman in the video then said, “Hello, how are you?” to the child and looked, verbalized, pointed, and/or verbalized at a joint attention target present or absent to the children’s visual field. All conversations were performed in French as the native language of participants.

The assessment of gaze following included 12 videos (4 seconds each in duration) of an actor with a neutral face first engaging in direct gaze and then shifting to 1 out of 3 objects. In 6 videos, the actor shifted his eyes and head to the target, and in 6 other videos, he only moved his eyes to the target. The same actors were engaged in another research protocol where their photo was shown for 5 seconds on half the screen next to an object. Other stimuli presented scenes with emotional valence extracted from cartoons in which the faces of the characters expressed an emotion that was either contingent or not contingent on the previous scene (total duration 5 minutes). Moreover, in all tests, the interstimulus interval lasted 2 seconds, during which a central crosshair was presented. The differences between the stimuli used included dynamic or static, human (male and female) or cartoon, and human or object. The counterbalancing of stimuli for participants and the number of participants included allowed the artificial intelligence and psychology teams to collaborate on the basis of this predefined research protocol. The results of these tests have been partly exploited, presented, and published [31-33].

### Data Transformation: Visualization of Eye-Tracking Scanpaths

The premise of this study is based on the learning of visual patterns included in eye-tracking scanpaths. Specifically, scanpaths are used as a means to compactly describe the gaze movements into a visual representation that can simplify the learning process. Further, the scanpaths were also used to visually encode the dynamics of eye motion using color gradients. To achieve this, we used the coordinates in eye-tracking records, which represented the participant's POG during the experiment runtime. Based on the change in POG over time, we were able to calculate the velocity of gaze movement. Subsequently, the scanpath and computed dynamics were transformed into images. For each participant, a set of images was constructed in 3 steps: (1) A line was drawn for each transition from (*x_t_, y_t_)* to (*x_t+1_, y_t+1_*), where *t* represents a point of time during the experiment. (2) The change in color across lines was used to visualize the movement dynamics. Through use of a grayscale spectrum, the color values were tuned based on the magnitude of velocity (ie, speed) with respect to time. (3) The images constructed were vertically mirrored since the origin was located at the bottom of the screen.

Images were constrained to contain approximately the same level of information. Specifically, a threshold was applied to limit the number of points to be drawn. The threshold was aimed to be high enough to sufficiently describe the pattern of gaze behavior. However, too-high values could increase the possibility of producing cluttered visualizations. Therefore, several tests were conducted to choose an appropriate value for the threshold. With a limit ranging from 100 to 150, images seemed to include fewer lines, which turned out to poorly discriminate the 2 classes of participants. Eventually, we decided to set the threshold to 200, which could largely strike an adequate balance and captured the key features of motion. The limit is was not a velocity threshold but a limitation to the amount of consecutive points drawn on any given scanpath image. We limited the dynamic values to a bound equal to a quarter of the diagonal of the screen because any higher movement would not be normal given the scenarios used for the capture.

**Figure 1:**
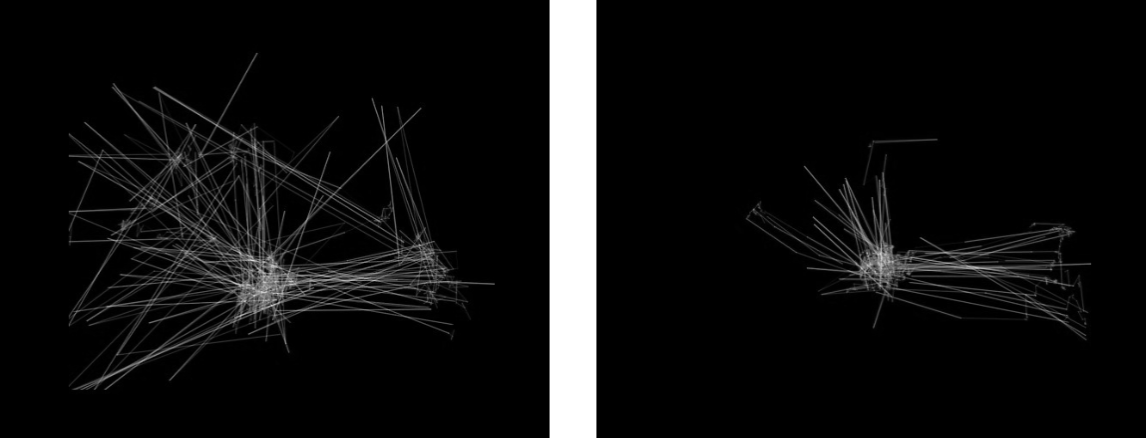
Visualization of eye-tracking scanpaths. The image on the left is from a participant diagnosed with autism spectrum disorder, while the one on the right is from a participant without the disorder.

The visualizations were produced using Python (Python Software Foundation) and a popular Matplotlib library [34]. The visualizations resulted in an image data set from the 59 participants who had viable data on an average of 15.19 different stimuli, allowing us to generate a total of 547 images (328 for non-ASD participants and 219 for those diagnosed with ASD), which corresponded to an average of 9.27 images per child (10.93 for non-ASD participants and 7.55 for those diagnosed with ASD). The default image dimensions were set as 640 x 480. The scanpath images were directly drawn from the raw data produced by the eye-tracking device. A more comprehensive presentation of the data set construction was elaborated upon in an earlier publication [27]. The data set was made freely available to be used by other studies investigating the potentials of eye tracking within the ASD context. Figure 1 presents 2 visualizations corresponding to participants with and without ASD.

### Data Preprocessing and Augmentation

Eye trackers can provide the POG coordinates on the screen. The coordinates were genuinely significant to implement our approach in terms of visualizing the gaze scanpath and computing its dynamics (eg, velocity). The eye-tracking records describe the category of movement and the POG for both eyes over time. To simplify the learning process, a set of image processing techniques was applied as follows. First, the black background was cropped from images as much as possible. The cropping was implemented using the OpenCV library. Second, all images were consistently scaled down to 256 x 256 dimensions. Resizing the images helped to reduce the problem of dimensionality by decreasing the number of features under consideration. The impact of resizing was also examined in the initial ML experiments.

Further, we applied image augmentation to produce variations of the scanpath images. Augmentation was recognized to generally improve the prediction accuracy in image classification applications [35,36]. The data set was augmented with an additional 2735 samples, where 5 synthetic samples were generated for each image. The data augmentation process was implemented using the Keras library [37], which includes an easy-to-use application programming interface for that purpose.

### Classification Model

The ML work described here falls into the category of supervised learning. The basic goal was to develop a binary classifier that could predict the class of participant (ie, ASD or non-ASD) based on the scanpath images. The classification model was implemented using an artificial neural network approach. Specifically, we designed a deep CNN.

CNNs typically include 3 categories of layers including convolutional layers, pooling layers, and fully connected layers [38]. The learning process goes through a series of convolutions and pooling, which break down the input image into a set of features maps. Convolutional layers initially attempt to extract features from the image through applying a convolutional kernel all over the image. Subsequently, pooling layers work on reducing the dimensions of feature maps extracted. Eventually, the output of this process usually feeds into a fully connected layer structure to produce the final prediction. In our case, the CNN model was composed of 4 convolutional layers, 4 pooling layers, and 2 fully connected layers. In addition, dropout layers were used, which help reduce the possibility of overfitting [39].

## Results

### Classification Accuracy

**Figure 2:**
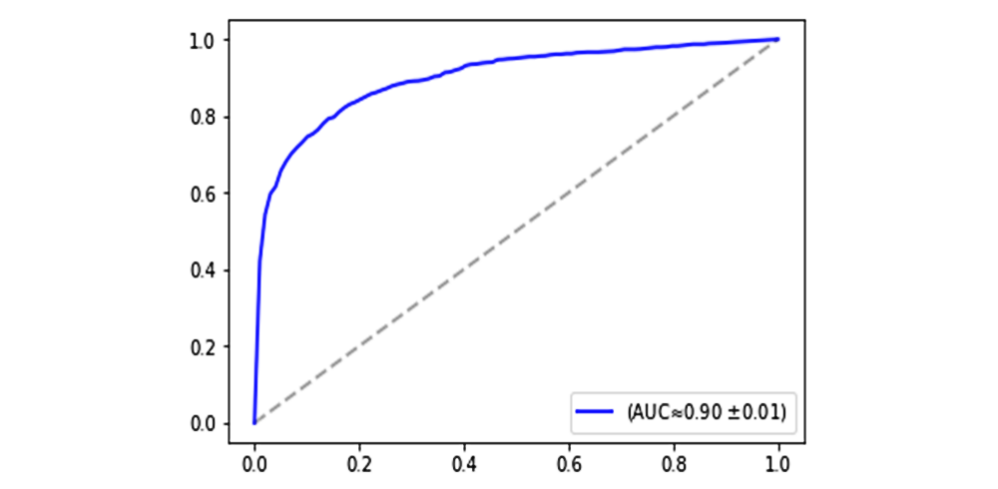
Receiver operating characteristic curve of the convolutional neural network model. AUC: area under the curve.

The classification accuracy was analyzed based on the receiver operating characteristic (ROC) curve. The ROC curve plotted the relationship between the true-positive rate and the false-positive rate across a full range of possible thresholds. Figure 2 plots the ROC curve of the CNN model. The figure also shows the approximate value of the area under the curve along with its standard deviation based on the 3-fold cross-validation. As it appears, the model could provide a notable prediction accuracy (≈90%), recall (ie, sensitivity; ≈83%), and precision (≈80%).

**Figure 3:**
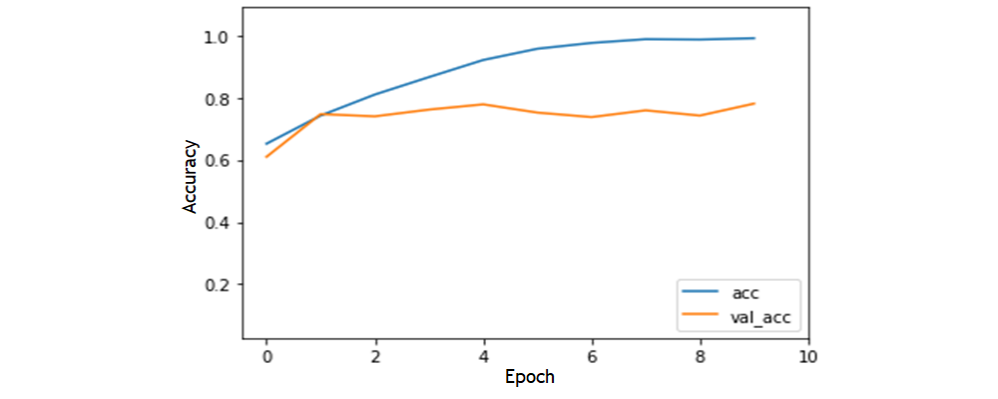
Model loss in the training and validation sets. acc: accuracy; val-acc: validation accuracy.

The model was implemented using the Keras library [37] with Python. The model was trained based on 3 rounds of cross-validation over 3 epochs. Training the model took ≈3 minutes using a single Tesla K80 GPU. Figure 3 demonstrates the model loss in training and validation over 3 epochs with 20% of the data set used for validation.

To further examine the model performance, the training and test sets were split based on the participants. The data set was split into training and test sets based on a 3-fold cross-validation using 3 stepwise procedures. First, the group of 59 participants were randomly split into 2 independent sets (ie, training and test), then the images were matched and loaded into the training and test sets based on the IDs of participants, and finally, these 2 steps were repeated for each round of the cross-validation process.

**Figure 4:**
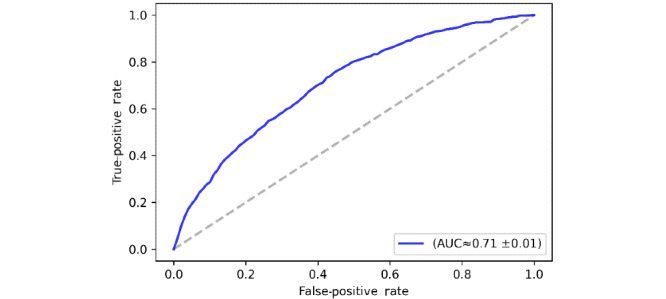
Receiver operating characteristic curve of all the data divided according to participants. AUC: area under the curve.

The features were extracted using the convolutional layers in the CNN model. The learning process goes through a series of convolutions and pooling, which break down the input image into a set of features maps. Convolutional layers initially attempt to extract features from the image through applying a convolutional kernel all over the image. Subsequently, pooling layers work on reducing the dimensions of feature maps extracted. Eventually, the output of this process usually feeds into a fully connected layer structure to produce the final prediction. Expectedly, the model performance declined as shown in Figure 4. The accuracy (≈71%) could still be viewed as promising given the relatively small data set.

### Correlation Analysis

This section serves as an integral part that supports the experimental results gained by our approach. Through statistical analysis, we attempted to explore possible correlations between the CARS score and the dynamics of eye movement in the eye-tracking scanpaths. Initially, the average velocity magnitude was calculated per image. In this way, the CARS scores of participants could be considered multiple times with respect to velocity. This could help mitigate the effect of outliers in eye-tracking experiments.

**Figure 5:**
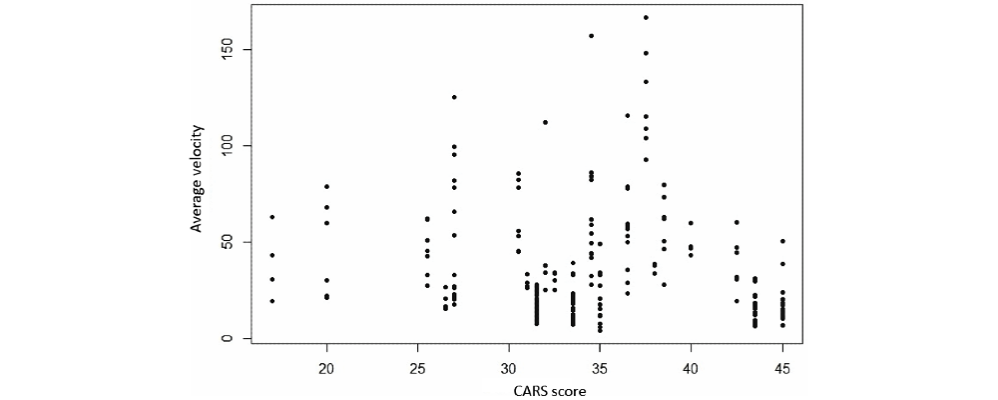
Average velocity depending on CARS value. Avg: average; CARS: Childhood Autism Rating Scale.

The patterns largely revealed the nonlinearity of the relationship between CARS scores and velocity. Therefore, standard correlation tests (eg, Pearson's *r*) would not be useful in such a case. Instead, we made use of the maximal information coefficient (MIC) [40]. The MIC score can describe the correlation between variable pairs regardless of a linear or nonlinear relationship. The score provided by MIC can be roughly considered as the coefficient of determination (*R*^2^). The MIC method has been embraced in a large number of studies to find correlations in complex data sets related to, for example, biology and genomics [41,42]. We used the Minerva R package [43], which greatly facilitated the computation of MIC. The MIC values (presented in Figure 5) suggested strong correlation between CARS and velocity (MIC= 0.79). The high correlation score result could partly validate the accuracy demonstrated by the classification model, whereas the velocity was visually encoded within the scanpath images.

### Demo Application

A demo application was developed to serve as a practical illustration of our approach. The application links the 3 components of eye-tracking, visualization, and ML together to support the diagnosis process of ASD.

The application goes through 3 steps as follows. First, the user is asked to upload the eye-tracking data. The data records should describe the coordinates of the viewer’s gaze into the screen along with the associated time. Second, the application produces a visualization of the eye-tracking scanpath. Eventually, the application calls the prediction web service, which returns the prediction from the trained classification model. Azure ML is employed to host the classification model and the Python implementation used to produce visualizations. The application can be accessed online by asking the authors for the URL link.

## Discussion

### Principal Results

This study demonstrated the strong potential of eye tracking as an objective tool for assisting ASD diagnosis. Indeed, abnormal eye gaze has been a hallmark characteristic of ASD [6,7]. Over several years, eye-tracking technology has been widely used to study attention impairment among individuals diagnosed with ASD [8,9,19,20]. In this paper, we introduced an additional dimension to the representation of eye-tracking scanpaths, and we demonstrated its effectiveness for training a classification model. In similar fashion to Frazier et al [21], we used static and dynamic stimuli including social and nonsocial images. However, adding nonsocial targets may be particularly important for increasing the relationship between nonsocial attention and ASD symptoms.

The empirical results provided a set of implications to be considered. First, the ML experiments confirmed the core idea behind our approach, which hinges on the visual representation of scanpaths. The classification accuracy indicated that scanpath visualizations were able to successfully pack the information of gaze motion and its underlying dynamics. This evidently translates into the validity of employing such visual patterns in order to diagnose individuals with ASD.

Equally important, the study brought further interesting insights into the features of autistic gaze. We provided a statistical analysis that revealed possible a correlation between the level of autism (ie, CARS) and the dynamic characteristics of eye motion (eg, velocity). The analysis can lend support to the findings of Vabalas and Freeth [17], which suggested that individuals with high autistic traits tend to have shorter and less frequent saccades compared to others with low autistic traits. However, the lack of a benchmark data set in the ASD literature makes it difficult to strictly compare our results to other ML approaches. A future larger project (with a cohort of children with ASD and typical children at different ages) should be considered and should analyze the socio-cognitive and cognitive profiles of children with ASD using eye tracking. The extensive literature on these different processes may be considered in connection with the study of gaze distinctiveness in children with ASD.

### Limitations

Even though the results presented in this study are promising, the following set of limitations should be highlighted. The primary limitation was the relatively small number of participants. In a future study, a data augmentation method for an ASD data set may be considered [44]. The interpretation of our results is limited by the fact that we did not have access to all the standardized test scores (ie, ADI and ASOS) used to clinically diagnose our study population. Also, the inclusion of ADI and ADOS scores could have provided further interpretation of the results. Another relevant issue of concern is the duration of video scenarios, which were relatively short. Perhaps longer scenarios might have allowed for a richer representation of the gaze behavior. Indeed, if the algorithm currently used and the age group of children in the model are limited for the moment, future work on a larger cohort will allow us to improve the study. In fact, despite limitations, we still believe this study can serve as the kernel for further interesting applications of the proposed approach.

### Conclusions

To conclude, the combination of eye tracking, visualization, and ML may hold considerable potential for the development of an objective tool to assist the diagnosis of ASD. These results can be used and new data analyzed to create a screening tool for health professionals Further, features related to the dynamics of eye movement can also be considered as candidate features for developing predictive models, and recently published deep neural network methodologies can be adapted to our model [45]. Eye-tracking measures which require limited technical expertise can be quickly managed during diagnostic interviews. Moreover, parents seem to have high acceptance of eye tracking as part of the clinical evaluation because the visual results are easier to understand than is the ADI cutoff, for example. In fact, for some parents, a lack of an objective measure can lead to delayed or diminished acceptance of the clinical diagnosis. However, some limitations may still delay the clinical adoption of eye tracking as an objective measure (eg, hardware and software costs), yet these issues can be reduced by consolidating the synergy between clinical structures and academic research.

## Abbreviations

ADI-R: Autism Diagnostic Interview-Revised
ADOS-G: Autism Diagnostic Observation Schedule-Generic
ASD: autism spectrum disorder
CARS: Childhood Autism Rating Scale
CNN: convolutional neural network
ESCS: Early Social Communication Scale
MIC: maximal information coefficient
ML: machine learning
POG: point of gaze
ROC: receiver operating characteristic

## References

[1] American Psychiatric Association (2013). Diagnostic and Statistical Manual of Mental Disorders DSM.

[2] Bosl W, Tierney A, Tager-Flusberg H, Nelson C (2011). EEG complexity as a biomarker for autism spectrum disorder risk. BMC Med.

[3] Frazier TW, Strauss M, Klingemier EW, Zetzer EE, Hardan AY, Eng C, Youngstrom EA (2017). A meta-analysis of gaze differences to social and nonsocial information between individuals with and without autism Meta-Analysis of Gaze Differences to Social and Nonsocial Information Between Individuals With and Without Autism. J Am Acad Child Adolesc Psychiatry.

[4] Shic F (2016). Eye tracking as a behavioral biomarker for psychiatric conditions: the road ahead. J Am Acad Child Adolesc Psychiatry.

[5] Jones E, Gliga T, Bedford R, Charman T, Johnson M (2014). Developmental pathways to autism: a review of prospective studies of infants at risk. Neurosci Biobehav Rev.

[6] Sepeta L, Tsuchiya N, Davies MS, Sigman M, Bookheimer SY, Dapretto M (2012). Abnormal social reward processing in autism as indexed by pupillary responses to happy faces. J Neurodev Disord.

[7] Kylliäinen Anneli, Wallace Simon, Coutanche Marc N, Leppänen Jukka M, Cusack James, Bailey Anthony J, Hietanen Jari K (2012). Affective-motivational brain responses to direct gaze in children with autism spectrum disorder. J Child Psychol Psychiatry.

[8] Kovarski K, Siwiaszczyk M, Malvy J, Batty M, Latinus M (2019). Faster eye movements in children with autism spectrum disorder. Autism Res.

[9] Król Magdalena Ewa, Król Michał (2019). A novel machine learning analysis of eye-tracking data reveals suboptimal visual information extraction from facial stimuli in individuals with autism. Neuropsychologia.

[10] Schopler E, Van BM, Wellman G, Love S (2010). Childhood Autism Rating Scale, Second Edition (CARS-2).

[11] Majaranta P, Bulling A, Fairclough SH, Gilleade K (2014). Eye tracking and eye-based human–computer interaction. Advances in Physiological Computing.

[12] Tullis T, Albert B (2013). Behavioral and physiological metrics. The User Experience: Collecting, Analyzing, And Presenting Usability Metrics.

[13] Benedetto S, Carbone A, Pedrotti M, Le Fevre K, Bey L, Baccino T (2015). Rapid serial visual presentation in reading: The case of Spritz. Computers in Human Behavior.

[14] Henderson J (2003). Human gaze control during real-world scene perception. Trends Cogn Sci.

[15] Jacob R, Barfield W, Furness TA (1995). Eye tracking in advanced interface design. Virtual Environments and Advanced Interface Design.

[16] Goldberg WA, Jarvis KL, Osann K, Laulhere TM, Straub C, Thomas E, Filipek P, Spence MA (2005). Brief report: early social communication behaviors in the younger siblings of children with autism. J Autism Dev Disord.

[17] Vabalas A, Freeth M (2016). Brief report: patterns of eye movements in face to face conversation are associated with autistic traits: evidence from a student sample. J Autism Dev Disord.

[18] Liberati A, Fadda R, Doneddu G, Congiu S, Javarone MA, Striano T, Chessa A (2017). A statistical physics perspective to understand social visual attention in autism spectrum disorder. Perception.

[19] Pierce K, Conant D, Hazin R, Stoner R, Desmond J (2011). Preference for geometric patterns early in life as a risk factor for autism. Arch Gen Psychiatry.

[20] Jones W, Klin A (2013). Attention to eyes is present but in decline in 2-6-month-old infants later diagnosed with autism. Nature.

[21] Frazier TW, Klingemier EW, Beukemann M, Speer L, Markowitz L, Parikh S, Wexberg S, Giuliano K, Schulte E, Delahunty C, Ahuja V, Eng C, Manos MJ, Hardan AY, Youngstrom EA, Strauss MS (2016). Development of an objective autism risk index using remote eye tracking. J Am Acad Child Adolesc Psychiatry.

[22] Samuel AL (2000). Some studies in machine learning using the game of checkers. IBM J. Res. & Dev.

[23] Pusiol G, Esteva A, Hall S, Frank M, Milstein A, Fei-fei L (2016). Vision-based classification of developmental disorders using eye-movements.

[24] Carette R, Cilia F, Dequen G, Bosche J, Guerin J, Vandromme L (2018). Automatic autism spectrum disorder detection thanks to eye-tracking and neural network-based approach. Internet Things Technol Healthc Autom Springer.

[25] Wei W, Liu Z, Huang L, Nebout A, Le MO (2019). Saliency prediction via multi-level features and deep supervision for children with autism spectrum disorder.

[26] Wang S, Jiang M, Duchesne X, Laugeson E, Kennedy D, Adolphs R, Zhao Q (2015). Atypical visual saliency in autism spectrum disorder quantified through model-based eye tracking. Neuron.

[27] Carette R, Elbattah M, Dequen G, Guerin J-L, Cilia F (2018). Visualization of eye-tracking patterns in autism spectrum disorder: method and dataset.

[28] Carette R, Elbattah M, Cilia F, Dequen G, Guérin J, Bosche J (2019). Learning to predict autism spectrum disorder based on the visual patterns of eye-tracking scanpaths.

[29] Rogé B (1989). Adaptation Française de l’échelle d’évaluation de l’autisme infantile (C.A.R.S.). Editions d’Applications Psychotechniques.

[30] Guidetti M, Tourrette C (2009). Evaluation de la Communication Sociale Précoce (ECSP). Eurotests. Parisurotests Editions.

[31] Cilia F, Aubry A, Le Driant B, Bourdin B, Vandromme L (2019). Visual exploration of dynamic or static joint attention bids in children with autism syndrome disorder. Front Psychol.

[32] Garry C, Cilia F, Landuré M, Aguillon EN, Rovira K, Brisson J (2017). Étude longitudinale de l'orientation sociale chez les enfants avec TSA d’âge préscolaire. Enfance.

[33] Cilia F, Aubry A, Bourdin B, Vandromme L (2019). Comment déterminer les zones d'intérêt visuelles sans a priori? Analyse des fixations d’enfants autistes en oculométrie. Revue de Neuropsychologie.

[34] Matplotlib: visualization with Python. Matplotlib.

[35] Xu Y, Jia R, Mou L, Li G, Chen Y, Lu Y, Jin Z (2016). Improved relation classification by deep recurrent neural networks with data augmentation.

[36] Wang J, Perez L (2017). The effectiveness of data augmentation in image classification using deep learning. Convolutional Neural Networks Vis. Recognit.

[37] Chollet F Keras. Github Repository.

[38] LeCun Y, Bengio Y, Hinton G (2015). Deep learning. Nature.

[39] Srivastava N, Hinton G, Krizhevsky A, Sutskever I, Salakhutdinov R (2014). Dropout: a simple way to prevent neural networks from overfitting. Journal of Machine Learning Research.

[40] Reshef DN, Reshef YA, Finucane HK, Grossman SR, McVean G, Turnbaugh PJ, Lander ES, Mitzenmacher M, Sabeti PC (2011). Detecting novel associations in large data sets. Science.

[41] Cho I, Blaser MJ (2012). The human microbiome: at the interface of health and disease. Nat Rev Genet.

[42] Maurice C, Haiser H, Turnbaugh P (2013). Xenobiotics shape the physiology and gene expression of the active human gut microbiome. Cell.

[43] Albanese D, Filosi M, Visintainer R, Riccadonna S, Jurman G, Furlanello C (2013). Minerva and minepy: a C engine for the MINE suite and its R, Python and MATLAB wrappers. Bioinformatics.

[44] Nebout A, Wei W, Liu Z, Huang L, Le MO (2019). Predicting saliency maps for ASD people.

[45] Ju Z, Gun L, Hussain A, Mahmud M, Ieracitano C (2020). A novel approach to shadow boundary detection based on an adaptive direction-tracking filter for brain-machine interface applications. Applied Sciences.

